# Effects of Neuroticism on Differences in Symptom Structure of Life Satisfaction and Depression-Anxiety among College Students: A Network Analysis

**DOI:** 10.3390/bs13080641

**Published:** 2023-08-01

**Authors:** Qihui Tang, Xiaoyan He, Liang Zhang, Xiangping Liu, Yanqiang Tao, Gang Liu

**Affiliations:** 1 Faculty of Psychology, Beijing Normal University, Beijing 100875, China; 2 Beijing Key Laboratory of Applied Experimental Psychology, National Demonstration Center for Experimental Psychology Education, Beijing 100875, China; 3 College Students’ Mental Health Education Center, Northeast Agricultural University, Harbin 150030, China; 4 Department of Psychiatry, Affiliated Nanjing Brain Hospital, Nanjing Medical University, Nanjing 210029, China

**Keywords:** depression, anxiety, life satisfaction, neuroticism, network analysis, college students

## Abstract

**Object:** Numerous studies show that depression and anxiety have an adverse effect on life satisfaction among college students. Moreover, neuroticism affects depression, anxiety, and life satisfaction. Comparing the low-neuroticism and high-neuroticism groups, the current study used network analysis to examine the relationship between depression, anxiety, and life satisfaction among college students. **Methods:** A sample consisted of 1233 college students from China who completed the Big Five Inventory-2 (BFI-2), Self-rating Anxiety Scale (SAS), Self-rating Depression Scale (SDS), and Satisfaction With Life Scale (SWLS).All students were divided into two groups according to levels of neuroticism. Depression-anxiety symptom networks and flow networks were formed. **Results:** “Insomnia” (SAS19) and “Sleep disturbance” (SDS4) are bridge symptoms of groups with varying neuroticism. In addition, compared to the group with low levels of neuroticism, the group with high levels of neuroticism showed more depression symptoms in bridge symptoms and greater global strength. Many depression-anxiety symptoms are negatively associated with life satisfaction, and “Emptiness” (SDS18) is an important symptom in the high-neuroticism group’s flow network. **Conclusion:** This study contributes to our understanding of the connection between depression, anxiety, neuroticism, and life satisfaction. In addition, the current study identified the essential symptoms to target in depression and anxiety intervention and life satisfaction enhancement among college students.

## 1. Introduction

With the development of positive psychology there is burgeoning literature focused on life satisfaction. Life satisfaction, a significant indicator of mental health [1], is an individual’s cognitive and global evaluation of their happiness with life [2]. Numerous studies have documented the relationship between life satisfaction and positive social, interpersonal, and intrapersonal outcomes [1,3]. Life satisfaction is also important for college students because college is a pivotal time when they face personal development challenges and must make numerous life-altering decisions [4,5]. For college students, literature shows that life satisfaction is positively associated with academic performance [6], self-esteem [7], physical activity [8], and mental health [9]. Thus, improvement of the level of life satisfaction of college students is conducive to their healthy growth.

To improve college students’ life satisfaction, we cannot ignore the effect of psychopathological variables on life satisfaction [1], particularly depression and anxiety. According to a study of 200 college students, those who experienced depression, anxiety, and stress were more susceptible to low life satisfaction [10]. Consistent with this study, abundant previous research has widely documented that depression and anxiety are negative predictors of life satisfaction [11,12,13]. Also worth noting is the prevalence of depression and anxiety among college students. Depression and anxiety are the most common mental problems among youth [14]. A meta-analysis involving 89 studies with 1,441,828 higher education students reported the prevalence of depressive symptoms and anxiety symptoms as 34% and 32%, respectively [15], a statistic that warrants attention. 

Considering the detrimental effect of depression and anxiety on life satisfaction and the high prevalence of depression and anxiety among college students, how to effectively intervene as well as improve life satisfaction for college students is a crucial research question warranting further exploration. However, despite the abundance of research examining the relationship between depression, anxiety, and life satisfaction, most studies are based on latent variable models that treat anxiety and depression as unidimensional variables [16,17] and may ignore the interaction between different symptoms of depression and anxiety [18]. Thus, in this study, we conduct a network analysis to examine the nuanced and comprehensive relationship between depression, anxiety, and life satisfaction by symptom level. Using network analysis, we can identify the bridge symptoms (i.e., symptoms that connect two mental disorders) [19] in the network, which can provide us with fresh insights to develop effective interventions that target the key symptoms in the network. This approach may enhance interventions for depression and anxiety and improve the life satisfaction of college students.

Within influential factors that affect depression and anxiety, personality variables have been identified as key factors [20,21]. Among the various personality theories, the Big Five personality theory is one of the most popular and widely used theories today and comprises the five factors of extraversion, openness, agreeableness, conscientiousness, and neuroticism [22,23]. Among these five factors, neuroticism is a special risk factor for a wide range of mental disorders [24]. Neuroticism is a stable personality trait that is defined as the tendency for people to experience negative emotions, such as anger, sad mood, and worry [25,26]. People with high level of neuroticism tend to be more sensitive to environmental stressors [27] and pay more attention to negative life events resulting in evocation of more negative emotions [28]. Higher levels of neuroticism are associated with higher risk of developing internalizing psychopathology [25,29] across the stages of childhood [30], adolescence [31], adulthood [28,32], and later life [33]. One study aimed at exploring the relationship between personality traits and psychological consequences of COVID-19, found that higher neuroticism was related to higher scores of stress and loneliness and that, although the other four factors of Big Five personality were significantly correlated with loneliness and stress, the effect sizes were small [34]. Previous research found that extraversion, openness, agreeableness, and conscientiousness are negatively associated with depression and anxiety while neuroticism showed a positive association [29,35,36] highlighting the peculiarities of neuroticism. Additionally, numerous studies have identified depression and anxiety susceptibility among people with high levels of neuroticism [37,38,39] which may be associated with lower life satisfaction [40,41]. Even though the negative relationship between neuroticism, depression, anxiety, and life satisfaction is well-established, the interaction between symptoms of depression, anxiety, and life satisfaction in groups with different levels of neuroticism has gone relatively unexplored. Therefore, to explore the relationship between depression, anxiety, and life satisfaction more comprehensively, we need to take neuroticism into account. 

Considering all the above, in the current study we applied network analysis to examine the relationship between depression, anxiety, and life satisfaction among college students, comparing the low-neuroticism group and the high-neuroticism group. The current study has three objectives. Firstly, we aim to examine the interaction between symptoms of anxiety and depression, identifying the bridge symptoms of the anxiety-depression network. Secondly, we constructed flow networks to investigate the direct or indirect relationship between the comorbidity of depression-anxiety and life satisfaction. Lastly, we compared the networks of the low-neuroticism group and high-neuroticism group to explore the difference between the two groups.

## 2. Method

### 2.1. Participants

Participants (*N* = 1238) in the current study were recruited from a university in Harbin, China. In 2021, participants completed the questionnaire through the Wenjuanxing online questionnaire platform (https://www.wjx.cn/, accessed on 9 June 2023). As Sawyer et al. [42] suggested defining the age range of 10–24 years as adolescence, in this study, five participants who did not meet the above age criteria were excluded and 1233 subjects (female = 618, Mean_age_ =18.29, SD_age_ = 0.78) were included in later analysis. Using the mean score of the neuroticism subscale (Mean_neuroticism_ = 32.38, SD_neuroticism_ = 8.26), we divided the subjects into two groups. A total of 685 subjects (female = 361, Mean_age_ = 18.30, SD_age_ = 0.75) with scores above the mean score on the neuroticism subscale divided into the high-neuroticism group, and 548 subjects (female = 257, Mean_age_ =18.30, SD_age_ = 0.80) were divided into the low-neuroticism group. The research was examined and approved by the ethics committee of Beijing Normal University (Reference number: 202112220084).

### 2.2. Measures

#### 2.2.1. Big Five Inventory-2 (BFI-2)

We used the sub-dimension of the neuroticism of BFI-2 in Chinese to measure the neuroticism of participants [43]. The sub-dimension of neuroticism contains 12 items, each scored on a 5-point Likert scale. Higher scores on the sub-dimension of neuroticism mean that the individuals show higher levels of neuroticism. The BFI-2 has good reliability and validity in China [43]. In the current study, the Cronbach’s α score of neuroticism sub-dimension was 0.872.

#### 2.2.2. Self-Rating Anxiety Scale (SAS)

The Self-rating Anxiety Scale (SAS), which was designed by Zung [44], was used in this research to measure the anxiety level of participants. The Chinese version is widely used and has solid reliability and validity [45]. This inventory has 20 items and each is scored on a 4-point Likert scale ranging from “1” (no or little time) to “4” (most or all of the time). After reversing the score of 5 items, the anxiety level of participants can be measured by the total score of all items, and a higher score means that the individual has a higher level of anxiety. This inventory shows a high level of internal consistency in the current study with a Cronbach’s α score of 0.890.

#### 2.2.3. Self-Rating Depression Scale (SDS)

The Self-rating Depression Scale (SDS) in Chinese was used to measure the level of depression [46,47]. This inventory has 20 items with 10 items that are reverse scored; each item is graded from “1” (no or little time) to “4” (most or all of the time). Participants with high total scores on this inventory may show higher levels of depression. The SDS has a Cronbach’s α score of 0.863, showing great internal consistency in the current study.

#### 2.2.4. Satisfaction with Life Scale (SWLS)

The SWLS was developed by Diener et al. [48] and was used to measure the level of life satisfaction. This scale contains five items and is graded from “1” to “7”. Each item score is added up to a total score for the SWLS with a higher total score in this inventory corresponding with higher levels of life satisfaction. The reliability and validity of the SWLS in China have been established by a previous study [49]. In this study, the Cronbach’s α score of SWLS was 0.878.

### 2.3. Data Analysis

In this study, all data were analyzed using *R* (Version 4.3.0) [50]. As a first step, we calculated the Means, standard deviations (SDs), skewness, and kurtosis of all SAS, SDS, and SWLS items. Additionally, we used the function *descrTable* of the *R* package *compareGroups* to compare the scores of items between the high-neuroticism group and low-neuroticism group [51]. 

Secondly, we used the *R* package *qgraph* [52] to estimate and visualize the anxiety-depression symptom network of all people—the low-neuroticism group and the high-neuroticism group, respectively. In this process, a Gaussian Graphical Model (GGM) was used to estimate associations between different symptoms and to construct the network between different symptoms [53]. Consequently, the Extended Bayesian Information Criteria (EBIC) and graphical least absolute shrinkage and selection operator (LASSO) models were used to regularize the GGM [54,55]. In the network, each node represents a symptom and each edge represents the regularized partial correlation between two nodes. Additionally, as to the edge, green lines mean that two nodes have positive correlations, red lines denote negative correlations, and thicker edges represent stronger relationships. 

To explore the significance of each symptom within the network, previous studies usually used the centrality index strength (i.e., the sum of the absolute edge weights connected to a specific node) which purports to be the most reliable centrality index [56,57]. However, Robinaugh et al. [58] suggested that strength may not be a reliable indicator when the network contains both positive and negative edges and that Expected Influence (EI) may prove a more reliable index. Thus, we used EI in the current study to measure the importance of each symptom within the network. We also used the *R* package *mgm* [57] to calculate the predictability (i.e., *R*^2^) of each node which represents the variance with which a node can be explained by neighboring nodes [59]. To identify the bridge symptoms in the networks—the overlapping symptoms of two disorders [60,61]—we calculated the bridge-expected influence (1-step) [58]. As in previous research, we set the standardized values of the bridge EI ≥ 1 as the criterion of bridge symptoms [19].

To compare the difference between the high-neuroticism group and low-neuroticism group, we used the *R* package *NetworkComparisonTest* [62] to conduct the Network Comparison Test (NCT). Additionally, we also conducted a bootstrap procedure to ensure that the networks constructed were robust via the *R* package *bootnet* [54]. Firstly, we conducted a nonparametric bootstrap, calculating the bootstrapped confidence intervals (95% CIs) to test the accuracy of edges in networks [54]. Secondly, we evaluated the differences between edge weights and centrality indices by utilizing bootstrapped difference tests. Lastly, we computed the correlation stability coefficient (*CS-C*) through case-dropping bootstrapping to examine the stability of the network model. The value of *CS-C* should be at least higher than 0.25 and preferably higher than 0.5 [54].

Finally, we used the function *flow* to plot a flow diagram and further explore the relationship between life satisfaction and other symptoms. The flow diagram places the node life satisfaction on the left side, then revealing direct and indirect relationships between life satisfaction and other symptoms.

## 3. Results

The Means and standard deviations (SD) of all items, and the results of *t*-tests between the high-neuroticism group and the low-neuroticism group are reported in Table 1. Table 1 shows that except for “Dyspnea” (SAS13) and Age, the scores of other symptoms show significant differences between the low-neuroticism group and the high-neuroticism group.

### 3.1. Network Structure

**Figure 1:**
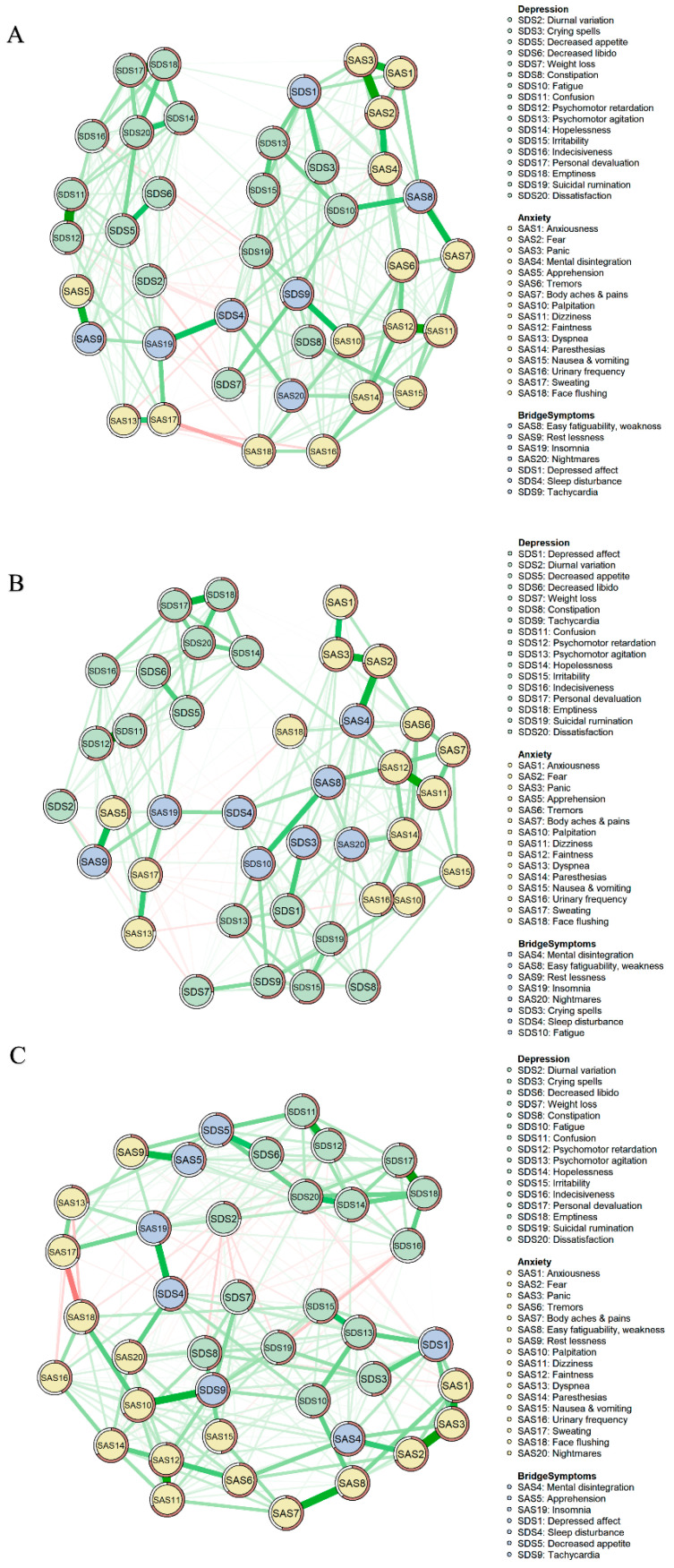
Network structures. (**A**), Network of all participants. (**B**), Network of the low-neuroticism group. (**C**), Network of the high-neuroticism group.

The anxiety-depression symptom networks of different groups are shown in Figure 1. Part A of Figure 1 shows the network of all participants. This network has 40 nodes and 780 edges with 330 non-zero (42.31%) edges. Among all edges in this network, “Confusion” (SDS11) and “Psychomotor retardation” (SDS12) show the strongest correlation, followed by the correlation between “Personal devaluation” (SDS17) and “Emptiness” (SDS18), and the correlation between “Panic” (SAS3) and “Fear” (SAS2) (refer to Table S1).

As shown in Part B of Figure 1, in the anxiety-depression network of the low-neuroticism group, there are 40 nodes and 276 non-zero edges (35.38%). The correlation between “Confusion” (SDS11) and “Psychomotor retardation” (SDS12) is the strongest one. We also found that the correlation between “Dizziness” (SAS11) and “Faintness” (SAS12) as well as the correlation between “Fear” (SAS2) and “Panic” (SAS3) displays strongly in this network (refer to Table S2). 

The anxiety-depression symptom network of the high-neuroticism group is shown in Part C of Figure 1. The network of the high-neuroticism group has 40 edges and 313 non-zero edges (40.13%). Among all edges in this network, the edge between “Personal devaluation” (SDS17) and “Emptiness” (SDS18), the edge between “Fear” (SAS2) and “Panic” (SAS3), and the edge between “Dizziness” (SAS11) and “Faintness” (SAS12) are the three strongest edges (refer to Table S3).

**Figure 2:**
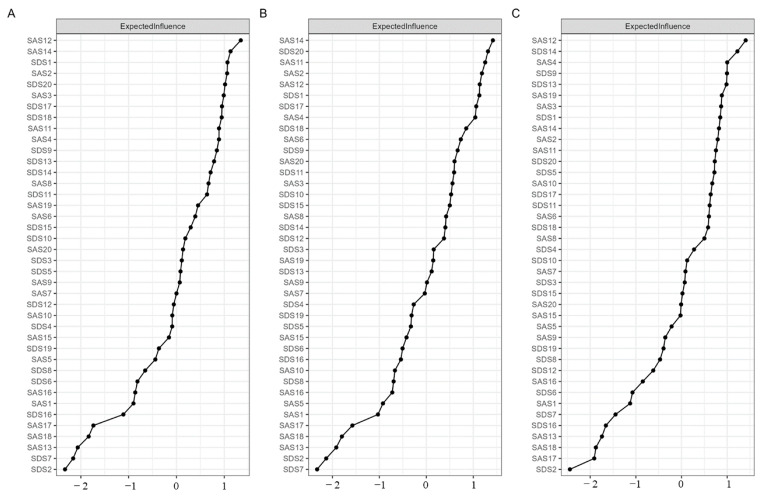
Centrality value. (**A**), Standardized EI among all participants. (**B**), Standardized EI of the low-neuroticism group. (**C**), Standardized EI of the high-neuroticism group.

Figure 2 shows the standardized EI of all symptoms for all participants (Part A)—the low-neuroticism group (Part B), and the high-neuroticism group (Part C). The symptoms with a high standardized EI may be considered central symptoms. In the network of all participants, the standardized EI of “Faintness” (SAS12), “Paresthesias” (SAS14), and “Depressed affect” (SDS1) ranked in the top three. Among these three nodes, “Faintness” (SAS12) was also the node with the highest standardized EI in the high-neuroticism group, and “Paresthesias” (SAS14) also had a high standardized EI in the low-neuroticism group. Additionally, in the low-neuroticism group network, “Dissatisfaction” (SDS20) and “Dizziness” (SAS11) also showed high standardized EI. In the high-neuroticism group, “Hopelessness” (SDS14) and “Mental disintegration” (SAS4) revealed high standardized EI. The results of standardized strength were shown in Figure S1.

**Figure 3:**
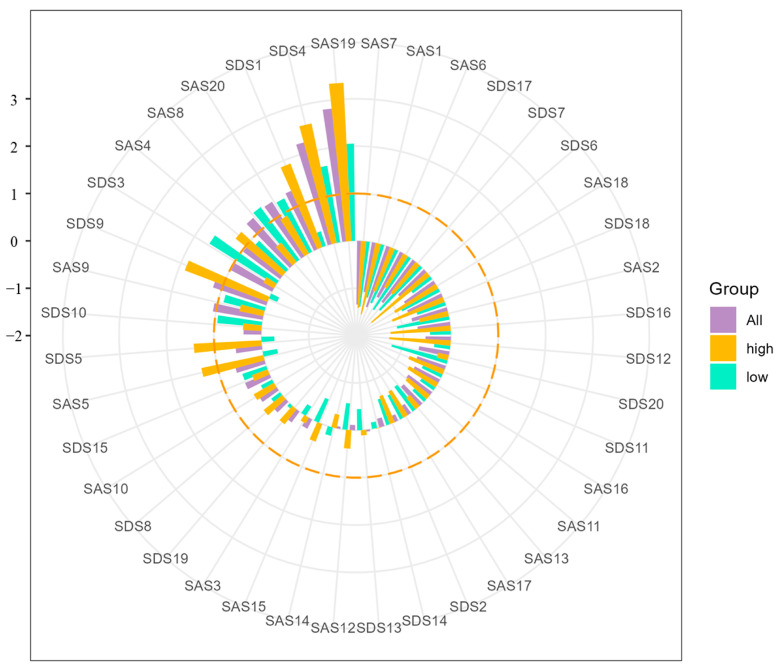
Bridge symptoms of all participants, low-neuroticism group, and high-neuroticism group (orange dotted line represents the bridge EI of 1 (standardized)).

The bridge symptoms of different networks are shown in Figure 3. Across three networks, “Insomnia” (SAS19) was the symptom with the highest standardized bridge EI. The standardized bridge EI “Sleep disturbance” (SDS4) was also ranked in the top three across three networks. Additionally, in the network of all participants, “Easy fatiguability & weakness” (SAS8), “Restlessness” (SAS9), “Nightmares” (SAS20), “Depressed affect” (SDS1), and “Tachycardia” (SDS9) were also bridge symptoms. In the network of low-neuroticism, other symptoms revealing high standardized bridge EI were “Mental disintegration” (SAS4), “Easy fatiguability & weakness” (SAS8), “Restlessness” (SAS9), “Nightmares” (SAS20), “Crying spells” (SDS3), and “Fatigue” (SDS10). In the network of high-neuroticism, “Mental disintegration” (SAS4), “Apprehension” (SAS5), “Depressed affect” (SDS1), “Decreased appetite” (SDS5), and “Tachycardia” (SDS9) were symptoms for which standardized bridge EI was higher than 1.

**Figure 4:**
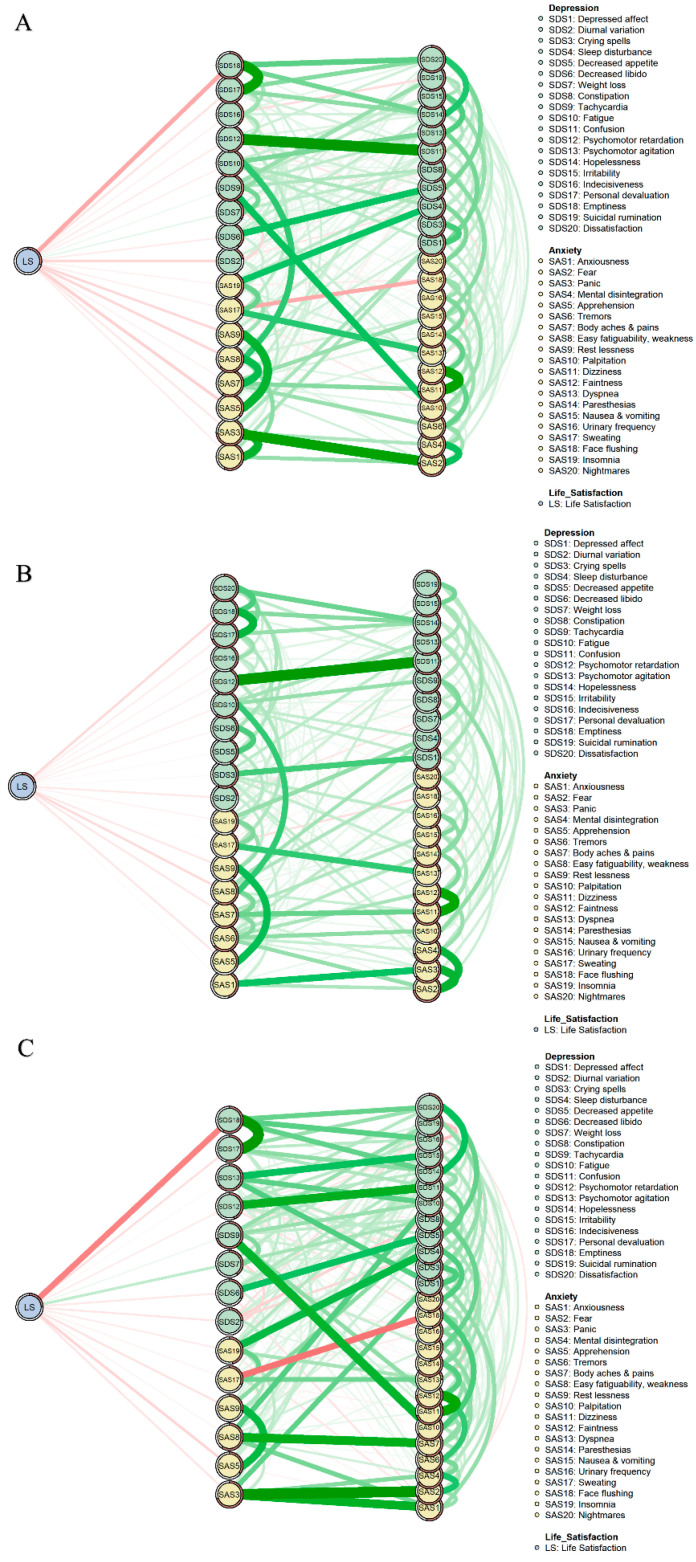
Flow network of life satisfaction. (**A**), Flow network of all participants. (**B**), Flow network of the low-neuroticism group. (**C**), Flow network of the high-neuroticism group.

The flow networks of all participants (Part A), the low-neuroticism group (Part B), and high-neuroticism group (Part C) are shown in Figure 4. In Figure 4 we find that, among all symptoms directly related to life satisfaction, “Emptiness” (SDS18) was the symptom most highly associated with life satisfaction for all three groups and a higher level of emptiness corresponded with lower life satisfaction.. Additionally, in the low-neuroticism group, the relationship between “Easy fatiguability & weakness” (SAS8) and life satisfaction was as strong as the relationship between “Emptiness” (SDS18) and life satisfaction. In the high-neuroticism group, “Weight loss” (SDS7) was the second strongest symptom associated with life satisfaction, and greater weight loss corresponded with greater life satisfaction. 

### 3.2. Network Comparison

**Figure 5:**
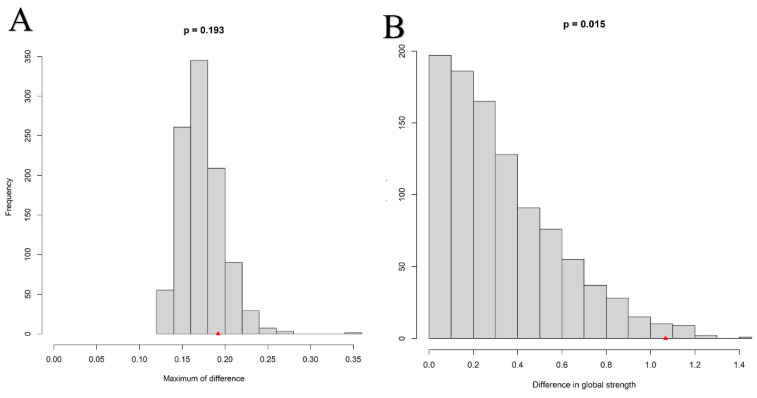
Network comparison between the low-neuroticism group and high-neuroticism group. (**A**) Edge weights invariance. (**B**) Global strength invariance.

The results of the network comparison are shown in Figure 5. Part A in Figure 5 shows the difference in the distribution of edge weights between the low-neuroticism group and the high-neuroticism group as non-significant (*M* = 0.192, *p* = 0.193). Part B in Figure 5 shows a significant difference between the global strength of the two groups (*S* = 1.068, *p* = 0.015).

### 3.3. Network Accuracy and Stability

To ensure the networks in the current study were accurate and stable, we conducted a bootstrap procedure. As Figure S2, the 95% CIs are narrow across the groups of all participants (Part A), the low-neuroticism group (Part B), and the high-neuroticism group (Part C). As seen in Figures S3 and S4, results show that most comparisons between edge weights and centrality indices were significant. Figure S5 reports the results of the case-dropping bootstrap, the *CS-C* values of all participants, the low-neuroticism group, and the high-neuroticism group are 0.7, 0.7, and 0.7, respectively.

## 4. Discussion

The current research conducted network analysis to investigate the relationship between depression, anxiety, and life satisfaction, factoring in level of neuroticism. Our study identified anxiety or depression symptoms associated with life satisfaction. Our study also identified the difference in global strength and bridge symptoms between the low-neuroticism group and the high-neuroticism group. Several significant findings require further discussion.

### 4.1. Bridge Symptoms of Depression-Anxiety Networks between the Low and High Neuroticism Groups

The bridge symptoms of the two groups reveal both similarities and differences. Specifically, the commonality between the two groups is that “Insomnia” (SAS19) and “Sleep disturbance” (SDS4) are bridge symptoms within both groups. These two symptoms are associated with sleep, an indispensable process in our daily life. Studies focused on sleep in adolescence document that sleep plays a crucial role in learning, attention, and cognitive processes [63]. Additionally, previous research also finds that sleep has a significant effect on mental health [64]. One study measured and followed up on the sleep quality and the mental health of college students, finding that poor sleep quality was associated with mental health problems (i.e., depression, anxiety, and stress) [65]. Consistent with this, research also finds that sleep loss is associated with heightened emotional reactivity to visual stimuli and changes in emotional memory processing [63,66,67], further supporting the notion that sleep is a significant indicator of mental health difficulties [68,69,70,71]. As for the relationship between sleep, depression, and anxiety, sleep problems are identified as common symptoms of both depression [72,73] and anxiety [74,75]. Furthermore, the relationship between depression, anxiety, and sleep may be bidirectional, which means that not only are people with anxiety and depression prone to having sleep problems, but people with sleep problems are also susceptible to developing other symptoms of anxiety and depression [76,77]. Presenting in the networks, sleep-related symptoms may serve as bridge symptoms in the anxiety-depression networks of the low-neuroticism group and the high-neuroticism group, consistent with the results of the current study.

The difference between the bridge symptoms of the two groups is that the high-neuroticism group reveals more symptoms of depression in bridge symptoms. Specifically, in the high-neuroticism group, there are four bridge symptoms related to depression and three bridge symptoms related to anxiety. In the low-neuroticism group, there are three bridge symptoms related to depression and five symptoms related to anxiety. Although anxiety and depression are both related to negative affect and stressful life events—their overlapping features—several studies also suggest that they have distinctive features [25,78]. As to their relationship with affect, compared to anxiety, depression is more associated with the absence of positive affect (i.e., happiness, interest, energy, and confidence) [79,80]. Supporting the difference between anxiety and depression, Khazanov and Ruscio [81] conducted a meta-analysis of longitudinal studies to examine the relationship between positive affect, anxiety, and depression and results showed that higher level of depression and anxiety is associated with less positive affect, but the relationship between positive affect and depression was greater. Additionally, neuroticism is also a factor that is related to positive affect, supported by the findings of Hisler et al. [82], who found that neuroticism has a negative influence on the average level of positive affect. Similarly, results of other research also documented the relationship between high levels of neuroticism and a decrease in positive affect [83]. Thus, we may infer that people with high levels of neuroticism, experiencing less positive affect, will be more susceptible to developing depression symptoms, and that the symptoms of depression may further activate other symptoms of depression or symptoms of anxiety [84]. However, these results must be interpreted with caution because, in the data, discrepancies still existed in the relationship between neuroticism and positive affect [82,85]. Future studies may further examine the effect of neuroticism on positive affect as well as the mechanisms by which neuroticism influences depression and anxiety.

### 4.2. Difference in Global Strength between the High-Neuroticism Group and Low-Neuroticism Group

Our study found that the global strength of the high-neuroticism group was higher than that of the low-neuroticism group. In other words, tighter connectivity existed between the symptoms in the high-neuroticism group, which may contribute to the vulnerability of individuals with high neuroticism to develop or maintain anxiety and depression symptoms [86]. Research by Smith et al. [87] found that neuroticism significantly predicted depression and anxiety and suggested that cognitive biases may explain the detrimental effect of neuroticism. Other studies likewise provide supportive evidence for the negative effects of neuroticism on anxiety and depression and are consistent with results of the current study [38,39]. Additionally, a review of neuroticism shows that neuroticism has its biological (i.e., autonomic nervous system) and psychological basis (i.e., cognitive processing of emotional information) [88] helping us understand the effect of neuroticism on depression and anxiety as well as the possible mechanisms behind this effect. According to these findings, at least, more studies are needed to investigate and to verify through which mechanisms neuroticism affects anxiety and depression, and to determine methods for the delivery of timely and effective interventions for highly neurotic college students with depression or anxiety. 

### 4.3. Flow Network between the Low and High Neuroticism Groups

As seen in the results of the flow network, in the high-neuroticism group and low-neuroticism group, most symptoms of depression and anxiety were directly or indirectly negatively correlated with life satisfaction. Significantly, our study identified the significant relationship between the symptoms of “Emptiness” (SDS18) and life satisfaction in the high-neuroticism group. “Emptiness” (SDS18) reflects whether the individual thinks “My life is meaningless” [89]. Similar to the findings of the current study, Ritchie et al. [90] investigated the relationship between neuroticism, meaninglessness, subjective well-being, and self-concept clarity and results showed that meaninglessness was positively associated with neuroticism while it was negatively related to life satisfaction. Furthermore, the implication of this finding is that among neurotic people with depression or anxiety, meaninglessness may be the key symptom to target to improve life satisfaction. Julom and de Guzmán [91] conducted a Logotherapy program, an existential therapy founded by Frankl [92], to intervene in the meaninglessness felt by paralyzed in-patients; the intervention ultimately decreased the meaninglessness felt by the experimental group. Consistent with prior research, several studies have also demonstrated the effectiveness of logotherapy in college students [93,94]. Notably, group logotherapy has been found to effectively alleviate depression and enhance life satisfaction among college students [93]. However, it is necessary to further investigate whether logotherapy can effectively address feelings of meaninglessness and enhance life satisfaction specifically in neurotic college students experiencing depression or anxiety.

These findings can be understood from the perspective of different facets of happiness, namely hedonic well-being and eudaimonic well-being [95,96]. Hedonic well-being principles encompass notions such as pleasure, enjoyment, and satisfaction, emphasizing the significance of life satisfaction and affective components [95,96]. In contrast, eudaimonic well-being focuses on optimal psychological functioning, encompassing concepts like personal growth, purpose in life, and a sense of autonomy [95]. Our study revealed a negative association between most depression and anxiety symptoms and scores on the Satisfaction with Life Scale (SWLS), which reflects hedonic well-being [97]. This result can potentially be explained by the physiological mechanisms underlying anxiety and depression, as changes in these mechanisms may influence an individual’s emotional perception [98]. Furthermore, the prominent role of “Emptiness” (SDS18) observed in the high-neuroticism group may indicate the impairment of eudaimonic well-being in neurotic individuals, as feelings of meaninglessness and depression are often associated with lower levels of eudaimonic well-being [99]. However, it is important to note that our study did not directly measure eudaimonic well-being, thus necessitating future research to further explore this hypothesis.

### 4.4. Limitations and Conclusions

Despite the significant findings, the present study has several weaknesses. Firstly, the current results are based on cross-sectional data. Therefore, the directed and causal relationship between symptoms of life satisfaction, neuroticism, depression, and anxiety is still largely unknown. Future studies may collect longitudinal data and use statistical methods such as cross-lagged panel network analysis (CLPN) to explore the directed or predictive relationship between symptoms and these variables. Secondly, the current study used self-report questionnaire to measure the level of life satisfaction, neuroticism, depression, and anxiety, which was mainly subjective. Future research should use behavioral experiments and use other objective neurophysiological indicators (i.e., fMRI, EEG) to test the results of the current study. Thirdly, the participants of this study were all from a university in China and results may not be generalizable to all cultures and all populations. Cross-cultural and cross-group research is needed to further examine the results of our study. The present research conducted network analysis and analyzed the relationship between life satisfaction and the comorbidity of depression and anxiety in groups with different levels of neuroticism. In this study, we found that the depression-anxiety networks between the high-neuroticism group and the low-neuroticism group have similarities; “Insomnia” (SAS19) and “Sleep disturbance” (SDS4) are bridge symptoms of both groups. As for the difference between the two groups, our study found that the high-neuroticism group has higher global strength and shows more symptoms of depression in bridge symptoms. Furthermore, in addition to finding that most symptoms of depression and anxiety have direct or indirect negative effects on life satisfaction, this study also identified “Emptiness” (SDS18) as an important symptom that is negatively associated with life satisfaction in the high-neuroticism group. Despite its limitations, the present study adds to the understanding of the relationship between depression, anxiety, neuroticism, and life satisfaction, generating fresh insights from the perspective of network analysis into the interventions for depression and anxiety as well as the improvement of life satisfaction among people with different levels of neuroticism.

## Figures and Tables

**Table 1:** Descriptive statistics for the low-neuroticism and high-neuroticism group and the *p*-values of *t*-tests between the two groups.

Labels	Symptoms	Items	Low-Neuroticism Group (*n* = 548)	High-Neuroticism Group (*n* = 685)	*p*
			Mean (SD)	Mean (SD)	
SAS1	Anxiousness	I feel more nervous and anxious than usual	1.56 (0.70)	2.30 (0.84)	<0.001
SAS2	Fear	I feel afraid for no reason at all	1.32 (0.61)	2.01 (0.86)	<0.001
SAS3	Panic	I get upset easily or feel panicky	1.39 (0.61)	2.14 (0.87)	<0.001
SAS4	Mental disintegration	I feel like I’m falling apart and going to pieces	1.26 (0.57)	1.86 (0.89)	<0.001
SAS5	Apprehension	I feel that everything is all right and nothing bad will happen	2.05 (1.00)	2.51 (0.87)	<0.001
SAS6	Tremors	My arms and legs shake and tremble	1.31 (0.59)	1.72 (0.81)	<0.001
SAS7	Body aches & pains	I am bothered by headaches neck and back pain	1.47 (0.74)	2.00 (0.89)	<0.001
SAS8	Easy fatiguability & weakness	I feel weak and get tired easily	1.51 (0.74)	2.20 (0.86)	<0.001
SAS9	Restlessness	I feel calm and can sit still easily	1.93 (0.91)	2.45 (0.88)	<0.001
SAS10	Palpitation	I can feel my heart beating fast	1.47 (0.71)	1.86 (0.79)	<0.001
SAS11	Dizziness	I am bothered by dizzy spells	1.34 (0.64)	1.80 (0.84)	<0.001
SAS12	Faintness	I have fainting spells or feel like it	1.26 (0.59)	1.69 (0.84)	<0.001
SAS13	Dyspnea	I can breathe in and out easily	2.64 (1.30)	2.65 (1.08)	0.937
SAS14	Paresthesias	I get feelings of numbness and tingling in my fingers and toes	1.22 (0.54)	1.68 (0.83)	<0.001
SAS15	Nausea & vomiting	I am bothered by stomachaches or indigestion	1.42 (0.72)	1.89 (0.91)	<0.001
SAS16	Urinary frequency	I have to empty my bladder often	1.42 (0.71)	1.88 (0.90)	<0.001
SAS17	Sweating	My hands are usually dry and warm	2.57 (1.09)	2.71 (0.96)	0.017
SAS18	Face flushing	My face gets hot and blushes	1.57 (0.78)	1.94 (0.84)	<0.001
SAS19	Insomnia	I fall asleep easily and get a good night’s rest	2.03 (1.10)	2.36 (0.98)	<0.001
SAS20	Nightmares	I have nightmares	1.35 (0.68)	1.78 (0.85)	<0.001
SDS1	Depressed affect	I feel down-hearted and blue	1.36 (0.65)	2.05 (0.84)	<0.001
SDS2	Diurnal variation	I feel the worst in the morning	2.49 (1.09)	2.75 (0.93)	<0.001
SDS3	Crying spells	I have crying spells or feel like it	1.37 (0.66)	1.93 (0.89)	<0.001
SDS4	Sleep disturbance	I have trouble sleeping at night	1.43 (0.76)	1.91 (0.95)	<0.001
SDS5	Decreased appetite	I eat less than usual	2.05 (1.11)	2.35 (0.99)	<0.001
SDS6	Decreased libido	I don’t feel as happy as before when I have close contact with the opposite sex	2.28 (1.08)	2.60 (0.96)	<0.001
SDS7	Weight loss	I notice that I am losing weight	1.67 (0.86)	1.90 (0.90)	<0.001
SDS8	Constipation	I’m troubled with constipation	1.39 (0.72)	1.76 (0.88)	<0.001
SDS9	Tachycardia	My heart beats faster than usual	1.36 (0.68)	1.77 (0.84)	<0.001
SDS10	Fatigue	I get tired for no reason	1.45 (0.73)	2.07 (0.88)	<0.001
SDS11	Confusion	I don’t have a clear mind as usual	2.08 (1.09)	2.47 (0.89)	<0.001
SDS12	Psychomotor retardation	I find it difficult to do what I used to do regularly	2.26 (1.07)	2.57 (0.89)	<0.001
SDS13	Psychomotor agitation	I am restless and can’t keep still	1.40 (0.70)	1.99 (0.88)	<0.001
SDS14	Hopelessness	I have no hope for the future	1.82 (1.02)	2.27 (0.93)	<0.001
SDS15	Irritability	I am more irritable than usual	1.38 (0.68)	2.00 (0.87)	<0.001
SDS16	Indecisiveness	I find it difficult to make decisions	2.37 (1.02)	2.68 (0.88)	<0.001
SDS17	Personal devaluation	I feel like a useless person and that no one needs me	1.90 (0.97)	2.40 (0.87)	<0.001
SDS18	Emptiness	My life is meaningless	1.88 (0.95)	2.43 (0.87)	<0.001
SDS19	Suicidal rumination	I feel that others would be better off if I were dead	1.34 (0.70)	1.77 (0.87)	<0.001
SDS20	Dissatisfaction	I’m no longer interested in what I’m usually interest in	1.89 (1.00)	2.27 (0.88)	<0.001
LS	Life Satisfaction		24.0 (6.12)	20.3 (5.63)	<0.001
Age			18.3 (0.80)	18.3 (0.75)	0.107
Neuroticism			25.2 (5.82)	38.2 (4.55)	<0.001

## Data Availability

The data presented in this study are available on request from the corresponding author.

## Supplementary Materials

The following supporting information can be downloaded at: https://www.mdpi.com/article/10.3390/bs13080641/s1.
