# Supplementary material for: Effects of Neuroticism on Differences in Symptom Structure of Life Satisfaction and Depression-Anxiety among College Students: A Network Analysis
Source: Behav Sci (Basel). 2023 Aug 1;13(8):641. doi: 10.3390/bs13080641 (PMC10451887; doi:10.3390/bs13080641)
Supplement: Supplementary file 1 [file behavsci-13-00641-s001.zip › behavsci-2495756-supplementary.pdf]

Table S1 The weight matrix of all participants

	SAS1	SAS2	SAS3	SAS4	SAS5	SAS6	SAS7	SAS8	SAS9	SAS10	SAS11	SAS12	SAS13	SAS14	SAS15	SAS16	SAS17	SAS18	SAS19	SAS20	SDS1	SDS2	SDS3	SDS4	SDS5	SDS6	SDS7	SDS8	SDS9	SDS10	SDS11	SDS12	SDS13	SDS14	SDS15	SDS16	SDS17	SDS18	SDS19	SDS20
SAS1	0	0.129	0.310	0	0	0	0	0.144	0	0	0	0	0	0	0	0.010	0	0.014	-0.024	0	0.110	0	0	0	0	0	0	-0.038	-0.009	0	0	0	0.013	0	0	0	0	0	0	0
SAS2	0.129	0	0.376	0.247	0	0.118	0.060	0	0.027	0.044	0	0	0	0	0	0	0	0.004	0	0.014	0.035	0	0.058	0	0	0	0	0	0	0	0	0	0.021	0	0	0	0.002	0	0	0
SAS3	0.310	0.376	0	0.101	0	0	0	0.057	0	0	0	0	0	0	0.007	0	0	0.014	0	0	0.106	0	0.025	0	0	0	0	0	0	0.034	0	0	0.032	0	0.046	0.0002	0	0.011	0	0
SAS4	0	0.247	0.101	0	0	0.132	0	0.044	0	0	0.017	0.077	0	0.018	0.029	0.053	0	0	0	0.017	0.078	0	0.051	0.044	0	0	0.016	0	0	0.005	0	0	0.029	0.027	0.031	0	0	0.014	0.066	0
SAS5	0	0	0	0	0	0	0	0	0.310	0	0	0	0.046	0	0	0	0.021	0	0.081	0	0.009	0.026	0.016	0	0.030	0.014	0	0	0	0	0	0	0	0.074	0	0.059	0.009	0.076	0	0
SAS6	0	0.118	0	0.132	0	0	0.124	0	0	0.124	0.034	0.179	0	0.123	0	0.030	0	0.043	0	0.032	0	0	0	0	0.012	0	0	0	0	0	0.004	0	0	0	0.013	0	0	0	0.006	0
SAS7	0	0.060	0	0	0	0.124	0	0.270	0	0.039	0.148	0	0	0.049	0.128	0.008	0	0.015	0	0	0	0	0.023	0.0003	0.002	0.010	0	0	0	0	0	0	0	0	0	0	0.001	0	0	0
SAS8	0.144	0	0.057	0.044	0	0	0.270	0	0	0.002	0.042	0	0	0	0.059	0	0	0	0	0.020	0.062	0.032	0	0.070	0	0	0	0	0	0.220	0	0	0	0	0.026	0	0	0	-0.003	0
SAS9	0	0.027	0	0	0.310	0	0	0	0	0	0	0	0.004	0.023	0	0	0.041	0	0.117	0	0	0.072	0	0	0.076	0.021	0	0	0	0	0.025	0.035	0.033	0.013	0.005	0.029	0.021	0	0	0.043
SAS10	0	0.044	0	0	0	0.124	0.039	0.002	0	0	0.093	0.081	-0.001	0.061	0	0.029	-0.035	0.125	0	0.012	0	0	0	0	0	-0.003	0	0	0.246	0	0	0	0	0	0.045	-0.004	0	0	0	0
SAS11	0	0	0	0.017	0	0.034	0.148	0.042	0	0.093	0	0.368	0	0.091	0.115	0	-0.015	0.016	0	0.048	0	0	0.042	0	0	0	0	0	0.020	0	0	0	0.041	0.012	0.022	0	0	0	0	0
SAS12	0	0	0	0.077	0	0.179	0	0	0	0.081	0.368	0	0	0.181	0	0.053	0	0	0	0.058	0	-0.012	0	0.004	0	0	0.029	0.014	0.065	0	0	0	0	0.044	0	0	0	0	0.055	0.010
SAS13	0	0	0	0	0.046	0	0	0	0.004	-0.001	0	0	0	0	0	-0.069	0.219	-0.026	0.057	0	0	0.017	0	-0.051	0.076	0	0	0	0	0	0.048	0.018	0	0	0	0.014	0	0	0	0.024
SAS14	0	0	0	0.018	0	0.123	0.049	0	0.023	0.061	0.091	0.181	0	0	0.052	0.154	0	0.063	0	0.121	0	-0.006	0.061	0.018	0	0	0.009	0.046	0.023	0	0	0	0.015	0	0	-0.004	0	0	0.055	0
SAS15	0	0	0.007	0.029	0	0	0.128	0.059	0	0	0.115	0	0	0.052	0	0.107	0	0.038	0	0.081	0.005	0	0.023	0.012	0	0	0.034	0.143	0	0.010	0	0	0	0	0	-0.002	0	0	0	0
SAS16	0.010	0	0	0.053	0	0.030	0.008	0	0	0.029	0	0.053	-0.069	0.154	0.107	0	-0.005	0.121	0	0.036	0	0	0.050	0.042	0	-0.017	0	0.067	0	0	0	0	0	0	0	0	0	0	0	0
SAS17	0	0	0	0	0.021	0	0	0	0.041	-0.035	-0.015	0	0.219	0	0	-0.005	0	-0.138	0.175	0	-0.009	0	0	0	0.016	0.025	-0.005	0	0	0	0	0.086	0	0	0	0.031	0.014	0	0	0.032
SAS18	0.014	0.004	0.014	0	0	0.043	0.015	0	0	0.125	0.016	0	-0.026	0.063	0.038	0.121	-0.138	0	-0.035	0.060	0.006	0	0	0	0	0	0	0.002	0.030	0.006	0	0	0	0	0.061	0	0.013	0	0	0
SAS19	-0.024	0	0	0	0.081	0	0	0	0.117	0	0	0	0.057	0	0	0	0.175	-0.035	0	0	0	0.074	0	0.242	0.089	0.068	-0.016	0	0	0	0.060	0.016	0	0.029	0	0.005	0.015	0.012	0	0.024
SAS20	0	0.014	0	0.017	0	0.032	0	0.020	0	0.012	0.048	0.058	0	0.121	0.081	0.036	0	0.060	0	0	0.011	0	0.012	0.155	0	0	0.002	0.035	0.016	0.003	0	-0.005	0.046	0	0.051	0	0	0	0.084	0
SDS1	0.110	0.035	0.106	0.078	0.009	0	0	0.062	0	0	0	0	0	0	0.005	0	-0.009	0.006	0	0.011	0	0	0.202	0.043	0	0	0.010	0.048	0	0.091	0	0	0.163	0	0.131	0	0	0.037	0	0
SDS2	0	0	0	0	0.026	0	0	0.032	0.072	0	0	-0.012	0.017	-0.006	0	0	0	0	0.074	0	0	0	0	-0.043	0.003	0.015	-0.066	-0.031	-0.024	0	0.063	0.055	0	0.028	0	0.062	0.059	0	-0.013	0
SDS3	0	0.058	0.025	0.051	0.016	0	0.023	0	0	0	0.042	0	0	0.061	0.023	0.050	0	0	0	0.012	0.202	0	0	0	0	0	0	0.087	0.028	0.052	0	0	0.073	0	0.014	0	0	0	0.090	0
SDS4	0	0	0	0.044	0	0	0.0003	0.070	0	0	0	0.004	-0.051	0.018	0.012	0.042	0	0	0.242	0.155	0.043	-0.043	0	0	0	-0.002	0.040	0.081	0.019	0.104	0	-0.043	0.066	0	0.018	-0.017	0	0	0.056	0

SDS5	0	0	0	0	0.030	0.012	0.002	0	0.076	0	0	0	0.076	0	0	0	0.016	0	0.089	0	0	0.003	0	0	0	0.238	0	0	0	0	0.084	0.062	0	0.013	0	0	0.038	0	0	0.160
SDS6	0	0	0	0	0.014	0	0.010	0	0.021	-0.003	0	0	0	0	0	-0.017	0.025	0	0.068	0	0	0.015	0	-0.002	0.238	0	-0.022	-0.022	0	0	0.051	0.013	0	0.076	-0.005	0.072	0.066	0.030	0	0.052
SDS7	0	0	0	0.016	0	0	0	0	0	0	0	0.029	0	0.009	0.034	0	-0.005	0	-0.016	0.002	0.010	-0.066	0	0.040	0	-0.022	0	0.083	0.166	0	0	-0.036	0.037	0	0.019	-0.002	0	-0.006	0.061	0
SDS8	-0.038	0	0	0	0	0	0	0	0	0	0	0.014	0	0.046	0.143	0.067	0	0.002	0	0.035	0.048	-0.031	0.087	0.081	0	-0.022	0.083	0	0.099	0.031	0	-0.016	0.012	0	0.011	0	0	0	0.068	0
SDS9	-0.009	0	0	0	0	0	0	0	0	0.246	0.020	0.065	0	0.023	0	0	0	0.030	0	0.016	0	-0.024	0.028	0.019	0	0	0.166	0.099	0	0.133	0	0	0.083	0	0.085	-0.025	0	0	0.128	0.002
SDS10	0	0	0.034	0.005	0	0	0	0.220	0	0	0	0	0	0	0.010	0	0	0.006	0	0.003	0.091	0	0.052	0.104	0	0	0	0.031	0.133	0	0.003	0	0.160	0	0.072	0	0	0	0	0
SDS11	0	0	0	0	0	0.004	0	0	0.025	0	0	0	0.048	0	0	0	0	0	0.060	0	0	0.063	0	0	0.084	0.051	0	0	0	0.003	0	0.403	0	0.104	0	0.003	0.062	0.035	0	0.090
SDS12	0	0	0	0	0	0	0	0	0.035	0	0	0	0.018	0	0	0	0.086	0	0.016	-0.005	0	0.055	0	-0.043	0.062	0.013	-0.036	-0.016	0	0	0.403	0	0	0.045	0	0.109	0.021	0.040	0	0.063
SDS13	0.013	0.021	0.032	0.029	0	0	0	0	0.033	0	0.041	0	0	0.015	0	0	0	0	0	0.046	0.163	0	0.073	0.066	0	0	0.037	0.012	0.083	0.160	0	0	0	0	0.195	0	0	0	0.052	0
SDS14	0	0	0	0.027	0.074	0	0	0	0.013	0	0.012	0.044	0	0	0	0	0	0	0.029	0	0	0.028	0	0	0.013	0.076	0	0	0	0	0.104	0.045	0	0	0	0.059	0.140	0.169	0	0.221
SDS15	0	0	0.046	0.031	0	0.013	0	0.026	0.005	0.045	0.022	0	0	0	0	0	0	0.061	0	0.051	0.131	0	0.014	0.018	0	-0.005	0.019	0.011	0.085	0.072	0	0	0.195	0	0	0	0	0	0.110	0
SDS16	0	0	0.0002	0	0.059	0	0	0	0.029	-0.004	0	0	0.014	-0.004	-0.002	0	0.031	0	0.005	0	0	0.062	0	-0.017	0	0.072	-0.002	0	-0.025	0	0.003	0.109	0	0.059	0	0	0.119	0.119	-0.057	0.038
SDS17	0	0.002	0	0	0.009	0	0.001	0	0.021	0	0	0	0	0	0	0	0.014	0.013	0.015	0	0	0.059	0	0	0.038	0.066	0	0	0	0	0.062	0.021	0	0.140	0	0.119	0	0.383	0	0.147
SDS18	0	0	0.011	0.014	0.076	0	0	0	0	0	0	0	0	0	0	0	0	0	0.012	0	0.037	0	0	0	0	0.030	-0.006	0	0	0	0.035	0.040	0	0.169	0	0.119	0.383	0	0	0.190
SDS19	0	0	0	0.066	0	0.006	0	-0.003	0	0	0	0.055	0	0.055	0	0	0	0	0	0.084	0	-0.013	0.090	0.056	0	0	0.061	0.068	0.128	0	0	0	0.052	0	0.110	-0.057	0	0	0	0.031
SDS20	0	0	0	0	0	0	0	0	0.043	0	0	0.010	0.024	0	0	0	0.032	0	0.024	0	0	0	0	0	0.160	0.052	0	0	0.002	0	0.090	0.063	0	0.221	0	0.038	0.147	0.190	0.031	0

Table S2 The weight matrix of low-neuroticism

	SAS1	SAS2	SAS3	SAS4	SAS5	SAS6	SAS7	SAS8	SAS9	SAS10	SAS11	SAS12	SAS13	SAS14	SAS15	SAS16	SAS17	SAS18	SAS19	SAS20	SDS1	SDS2	SDS3	SDS4	SDS5	SDS6	SDS7	SDS8	SDS9	SDS10	SDS11	SDS12	SDS13	SDS14	SDS15	SDS16	SDS17	SDS18	SDS19	SDS20
SAS1	0	0.121	0.283	0.008	0	0.010	0.002	0.079	0	0.034	0	0	0	0	0	0	0	0.034	0	0	0.023	0	0.035	0	0	0	0	0	0	0	0	0	0	0.004	0	0	0	0	0	0
SAS2	0.121	0	0.359	0.346	0	0.153	0.027	0	0	0.004	0	0	0	0	0	0	0	0	0	0.078	0.038	0	0	0	0	0	0.006	0	0.010	0	0	0	0	0.007	0	0	0	0.007	0	0
SAS3	0.283	0.359	0	0	0	0.055	0.070	0	0.013	0	0.035	0	0	0	0	0	0	0	0	0	0	0	0.071	0	0	0	0	0	0	0.038	0	0	0.076	0	0	0	0	0.008	0	0
SAS4	0.008	0.346	0	0	0	0.084	0	0.004	0	0	0	0.148	0	0.066	0.028	0.025	0	0.040	0	0.028	0.017	0	0.089	0.080	0	0	0.004	0	0	0	0	0	0	0	0.054	0	0	0	0.100	0
SAS5	0	0	0	0	0	0	0	0	0.312	0	0	0	0.056	0	0	0	0.007	0	0.078	0.023	0	0.009	0	0	0	0	0	0	0	0	0	0	0	0.079	0	0.036	0	0.057	0	0
SAS6	0.010	0.153	0.055	0.084	0	0	0.176	0	0.017	0.163	0	0.125	0	0.112	0	0.002	0	0.102	0	0	0.041	0	0	0	0	0	0	0	0	0	0.010	0	0	0	0	0	0	0	0	0
SAS7	0.002	0.027	0.070	0	0	0.176	0	0.194	0	0.031	0.208	0	0	0.003	0.116	0	0	0.008	0	0	0.004	0	0.023	0	0	0	0	0	0	0	0	0	0	0	0	0	0.005	0	0	0
SAS8	0.079	0	0	0.004	0	0	0.194	0	0	0.016	0.084	0	0	0	0.035	0.023	0	0	0	0.121	0.020	0	0	0.138	0.004	0	0	0	0	0.256	0	0	0	0	0	0	0	0	0	0
SAS9	0	0	0.013	0	0.312	0.017	0	0	0	0	0	0	0.031	0.008	0	0	0	0	0.150	0	0	0.096	0	0	0.019	0.028	0	0	0	0.041	0.026	0.035	0	0	0.004	0.039	0.001	0.021	0	0.038
SAS10	0.034	0.004	0	0	0	0.163	0.031	0.016	0	0	0.050	0.068	0	0.073	0	0	-0.024	0.040	0	0	0.022	0	0	0	0	0	0	0	0.158	0	0	0	0	0	0.082	0	0	0	0	0
SAS11	0	0	0.035	0	0	0	0.208	0.084	0	0.050	0	0.419	0	0.038	0.131	0	0	0.002	0	0.027	0.007	0	0.051	0	0	0	0	0	0	0	0	0	0.034	0	0.052	0	0	0	0.033	0
SAS12	0	0	0	0.148	0	0.125	0	0	0	0.068	0.419	0	0	0.178	0	0.061	0	0	0	0	0.002	0	0	0	0	0	0.043	0	0	0	0	0	0	0.101	0	0	0	0	0	0
SAS13	0	0	0	0	0.056	0	0	0	0.031	0	0	0	0	0	0	-0.055	0.254	0	0	0	0	0	0	-0.037	0.046	0	0	0	0	0	0.048	0.023	0	0.015	0	0.026	0	0	0	0.013
SAS14	0	0	0	0.066	0	0.112	0.003	0	0.008	0.073	0.038	0.178	0	0	0.092	0.164	0	0.059	0	0.167	0.007	0	0.089	0	0	0	0.003	0	0.016	0	0	0	0.055	0	0.023	0	0	0.001	0.057	0
SAS15	0	0	0	0.028	0	0	0.116	0.035	0	0	0.131	0	0	0.092	0	0.176	0	0	0	0.046	0	0	0.008	0.030	0	0	0	0.087	0	0	0	0	0	0	0.016	0	0	0.009	0	0
SAS16	0	0	0	0.025	0	0.002	0	0.023	0	0	0	0.061	-0.055	0.164	0.176	0	0	0.075	0	0.055	0	0	0.099	0	0	0	0.0005	0.077	0	0	0	0	0	0	0	0	0	0	0	0
SAS17	0	0	0	0	0.007	0	0	0	0	-0.024	0	0	0.254	0	0	0	0	-0.065	0.185	0	-0.020	0	-0.010	0	0	0.049	-0.031	0	-0.008	0	0	0.119	-0.026	0	0	0.062	0	0.006	0	0.003
SAS18	0.034	0	0	0.040	0	0.102	0.008	0	0	0.040	0.002	0	0	0.059	0	0.075	-0.065	0	-0.014	0.070	0	0	0	0	0	0	0.017	0.015	0.022	0	0	0	0.023	0	0	0	0.022	0	0	0
SAS19	0	0	0	0	0.078	0	0	0	0.150	0	0	0	0	0	0	0	0.185	-0.014	0	0	0	0.039	0	0.172	0.110	0.077	0	0	0	0	0.077	0	0	0	0	0	0.009	0.026	0.001	0
SAS20	0	0.078	0	0.028	0.023	0	0	0.121	0	0	0.027	0	0	0.167	0.046	0.055	0	0.070	0	0	0.110	0	0	0.070	0.008	0	0.003	0.078	0	0.021	0	0	0	0	0.068	0	0	0	0.044	0
SDS1	0.023	0.038	0	0.017	0	0.041	0.004	0.020	0	0.022	0.007	0.002	0	0.007	0	0	-0.020	0	0	0.110	0	0	0.244	0.066	0	0	0.012	0.090	0.092	0.092	0	0	0.125	0	0.131	0	0	0.020	0	0
SDS2	0	0	0	0	0.009	0	0	0	0.096	0	0	0	0	0	0	0	0	0	0.039	0	0	0	0	0	0.031	0	-0.055	0	-0.004	-0.002	0.054	0.037	0	0	0	0.083	0.028	0.009	0	0.045
SDS3	0.035	0	0.071	0.089	0	0	0.023	0	0	0	0.051	0	0	0.089	0.008	0.099	-0.010	0	0	0	0.244	0	0	0.001	0	0	0.006	0.051	0	0.046	0	0	0.099	0	0	0	0	0	0.011	0
SDS4	0	0	0	0.080	0	0	0	0.138	0	0	0	0	-0.037	0	0.030	0	0	0	0.172	0.070	0.066	0	0.001	0	0	0	0.012	0.063	0	0.093	0	-0.015	0.059	0	0.011	-0.011	0	0	0.079	0
SDS5	0	0	0	0	0	0	0	0.004	0.019	0	0	0	0.046	0	0	0	0	0	0.110	0.008	0	0.031	0	0	0	0.239	0	0	0	0	0.013	0.060	0	0.034	0	0	0.118	0	0	0.116

SDS6	0	0	0	0	0	0	0	0	0.028	0	0	0	0	0	0	0	0.049	0	0.077	0	0	0	0	0	0.239	0	-0.017	0	0	0	0.029	0.008	0	0.053	0	0.074	0.029	0.059	0	0.128
SDS7	0	0.006	0	0.004	0	0	0	0	0	0	0	0.043	0	0.003	0	0.0005	-0.031	0.017	0	0.003	0.012	-0.055	0.006	0.012	0	-0.017	0	0.048	0.182	0.021	0	-0.025	0.007	0	0.061	0	0	0	0.026	0
SDS8	0	0	0	0	0	0	0	0	0	0	0	0	0	0	0.087	0.077	0	0.015	0	0.078	0.090	0	0.051	0.063	0	0	0.048	0	0.096	0.037	0	0	0.005	0	0	0	0	0	0.062	0
SDS9	0	0.010	0	0	0	0	0	0	0	0.158	0	0	0	0.016	0	0	-0.008	0.022	0	0	0.092	-0.004	0	0	0	0	0.182	0.096	0	0.152	0	0	0.104	0	0.103	0	-0.005	0	0.114	0
SDS10	0	0	0.038	0	0	0	0	0.256	0.041	0	0	0	0	0	0	0	0	0	0	0.021	0.092	-0.002	0.046	0.093	0	0	0.021	0.037	0.152	0	0	0	0.135	0	0.070	0	0	0	0	0
SDS11	0	0	0	0	0	0.010	0	0	0.026	0	0	0	0.048	0	0	0	0	0	0.077	0	0	0.054	0	0	0.013	0.029	0	0	0	0	0	0.480	0	0.094	0	0.032	0.004	0.051	0	0.098
SDS12	0	0	0	0	0	0	0	0	0.035	0	0	0	0.023	0	0	0	0.119	0	0	0	0	0.037	0	-0.015	0.060	0.008	-0.025	0	0	0	0.480	0	0	0.005	0	0.101	0.090	0.014	0	0.033
SDS13	0	0	0.076	0	0	0	0	0	0	0	0.034	0	0	0.055	0	0	-0.026	0.023	0	0	0.125	0	0.099	0.059	0	0	0.007	0.005	0.104	0.135	0	0	0	0	0.133	0	0	0	0.073	0
SDS14	0.004	0.007	0	0	0.079	0	0	0	0	0	0	0.101	0.015	0	0	0	0	0	0	0	0	0	0	0	0.034	0.053	0	0	0	0	0.094	0.005	0	0	0	0.054	0.149	0.174	0	0.204
SDS15	0	0	0	0.054	0	0	0	0	0.004	0.082	0.052	0	0	0.023	0.016	0	0	0	0	0.068	0.131	0	0	0.011	0	0	0.061	0	0.103	0.070	0	0	0.133	0	0	-0.010	0	0	0.196	0
SDS16	0	0	0	0	0.036	0	0	0	0.039	0	0	0	0.026	0	0	0	0.062	0	0	0	0	0.083	0	-0.011	0	0.074	0	0	0	0	0.032	0.101	0	0.054	-0.010	0	0.147	0.032	0	0.083
SDS17	0	0	0	0	0	0	0.005	0	0.001	0	0	0	0	0	0	0	0	0.022	0.009	0	0	0.028	0	0	0.118	0.029	0	0	-0.005	0	0.004	0.090	0	0.149	0	0.147	0	0.346	0	0.185
SDS18	0	0.007	0.008	0	0.057	0	0	0	0.021	0	0	0	0	0.001	0.009	0	0.006	0	0.026	0	0.020	0.009	0	0	0	0.059	0	0	0	0	0.051	0.014	0	0.174	0	0.032	0.346	0	0	0.236
SDS19	0	0	0	0.100	0	0	0	0	0	0	0.033	0	0	0.057	0	0	0	0	0.001	0.044	0	0	0.011	0.079	0	0	0.026	0.062	0.114	0	0	0	0.073	0	0.196	0	0	0	0	0.005
SDS20	0	0	0	0	0	0	0	0	0.038	0	0	0	0.013	0	0	0	0.003	0	0	0	0	0.045	0	0	0.116	0.128	0	0	0	0	0.098	0.033	0	0.204	0	0.083	0.185	0.236	0.005	0

Table S3 The weight matrix of high-neuroticism

	SAS1	SAS2	SAS3	SAS4	SAS5	SAS6	SAS7	SAS8	SAS9	SAS10	SAS11	SAS12	SAS13	SAS14	SAS15	SAS16	SAS17	SAS18	SAS19	SAS20	SDS1	SDS2	SDS3	SDS4	SDS5	SDS6	SDS7	SDS8	SDS9	SDS10	SDS11	SDS12	SDS13	SDS14	SDS15	SDS16	SDS17	SDS18	SDS19	SDS20
SAS1	0	0.116	0.284	0	-0.016	0	0	0.149	-0.017	0	0	0	0	0	0	0	0	0	-0.033	0	0.121	0	0	0	0	-0.040	0	-0.038	-0.018	0	0	0	0.022	-0.005	0	0	0	0	0	-0.033
SAS2	0.116	0	0.363	0.202	0.003	0.100	0.050	0.006	0	0.060	0	0	0	0	0	0	0	0.017	0	0	0.010	0	0.078	0	0	0	0	0	0	0.023	0	0	0.026	0	0	0	0	0	0	0
SAS3	0.284	0.363	0	0.133	0	0	0	0.071	0	0	0	0	0	0	0	0	-0.019	0	0	0	0.165	0	0.002	0	0	0	0	0	-0.005	0.012	0	0	0.006	0	0.062	0	0	0	0	0
SAS4	0	0.202	0.133	0	0.001	0.143	0	0.056	0	0.010	0.028	0.057	0	0.002	0.011	0.053	0	0	0	0.018	0.088	0	0.045	0.030	0	0	0.028	0	0	0.037	0	0	0.047	0.050	0.006	0	0.002	0.018	0.052	0
SAS5	-0.016	0.003	0	0.001	0	0	0	0	0.258	0	0	0	0.024	0	0	0	0.016	-0.015	0.077	0	0.013	0.013	0.009	0	0.064	0.020	0	0	0	0	0.034	0.039	0	0.040	0	0.067	0.069	0.044	0	0
SAS6	0	0.100	0	0.143	0	0	0.094	0.014	0	0.090	0.049	0.194	0	0.124	0.029	0.042	0	0	0	0.032	0	0	0	0	0.027	0	0	0	0	0	0	0	0	0	0.044	0	0	0	0.016	0
SAS7	0	0.050	0	0	0	0.094	0	0.294	0	0.041	0.117	0	0	0.078	0.123	0.016	0	0.006	0	0	0	0	0.020	0.008	0	0	0	0	0	0	0	0	0	0	0	0	0	0	0	0
SAS8	0.149	0.006	0.071	0.056	0	0.014	0.294	0	0	0	0.030	0	0	0	0.053	0	0	0	0	0	0.046	0	0	0.035	0	0	0	0	0	0.174	0	0	0.013	0	0.028	0	0	0	0	0
SAS9	-0.017	0	0	0	0.258	0	0	0	0	0	0	0	0	0.011	0	0	0.082	0	0.089	0	0	0.017	0	0	0.129	0.009	0	0	0	0	0	0.018	0.021	0.040	0	0.007	0.006	0	0	0.048
SAS10	0	0.060	0	0.010	0	0.090	0.041	0	0	0	0.104	0.081	0	0.054	0.018	0.060	-0.012	0.180	0	0.041	0	0	0	0.017	0	0	0	0	0.276	0	0	0	0	0	0.012	-0.013	0	0	0	0
SAS11	0	0	0	0.028	0	0.049	0.117	0.030	0	0.104	0	0.317	0	0.116	0.090	0	-0.020	0.022	0	0.048	0	0	0.033	0	0	0	0	0	0.053	0	0	0	0.041	0.014	0.001	0	0	0	0	0
SAS12	0	0	0	0.057	0	0.194	0	0	0	0.081	0.317	0	0	0.175	0	0.042	0	0	0	0.090	0	-0.008	0	0.001	0	0	0.014	0.023	0.123	0	0	0	0	0	0	0	0	0	0.077	0.049
SAS13	0	0	0	0	0.024	0	0	0	0	0	0	0	0	0	0	-0.060	0.151	-0.085	0.101	0	0	0.036	0	-0.034	0.102	0	0	0	0	0	0.040	0.010	0	0	0	0	0	0	0	0.028
SAS14	0	0	0	0.002	0	0.124	0.078	0	0.011	0.054	0.116	0.175	0	0	0.019	0.137	0	0.062	0	0.101	0	-0.009	0.044	0.021	0	-0.013	0.011	0.105	0	0	0	0	0	0	0	-0.018	0	0	0.043	0
SAS15	0	0	0	0.011	0	0.029	0.123	0.053	0	0.018	0.090	0	0	0.019	0	0.054	0	0.051	0	0.087	0.044	0	0.008	0	0	0	0.066	0.157	0.018	0.013	0	0	0	0	0	-0.025	0	0	0	0
SAS16	0	0	0	0.053	0	0.042	0.016	0	0	0.060	0	0.042	-0.060	0.137	0.054	0	-0.030	0.122	0	0.013	0	0	0.012	0.075	0	-0.026	0	0.048	0.018	0	0	0	0	0	0	0	0	0	0	0
SAS17	0	0	-0.019	0	0.016	0	0	0	0.082	-0.012	-0.020	0	0.151	0	0	-0.030	0	-0.200	0.159	0	0	0.007	0	0	0.039	0	0	0	0	0	0.010	0.021	0	0	0	0	0.018	0	0	0.041
SAS18	0	0.017	0	0	-0.015	0	0.006	0	0	0.180	0.022	0	-0.085	0.062	0.051	0.122	-0.200	0	-0.002	0.037	0.001	0	0	0	0	0	0	0	0.014	0.020	0	-0.041	0	0	0.086	0	0	0	0	0
SAS19	-0.033	0	0	0	0.077	0	0	0	0.089	0	0	0	0.101	0	0	0	0.159	-0.002	0	0	0	0.083	0	0.255	0.064	0.055	-0.010	0	0	0	0.031	0.019	0	0.087	0	0.032	0.016	0	0	0.059
SAS20	0	0	0	0.018	0	0.032	0	0	0	0.041	0.048	0.090	0	0.101	0.087	0.013	0	0.037	0	0	0	-0.002	0.009	0.176	0	0	0	0	0.005	0	0	-0.023	0.063	0	0.033	-0.003	0	0	0.094	0
SDS1	0.121	0.010	0.165	0.088	0.013	0	0	0.046	0	0	0	0	0	0	0.044	0	0	0.001	0	0	0	0	0.179	0.042	0	0	0.003	0.011	0	0.068	0	0	0.176	0	0.093	0	0	0.005	0.007	0
SDS2	0	0	0	0	0.013	0	0	0	0.017	0	0	-0.008	0.036	-0.009	0	0	0.007	0	0.083	-0.002	0	0	-0.007	-0.060	0	0.022	-0.061	-0.065	-0.022	0	0.056	0.054	0	0.052	0	0.004	0.045	0	-0.048	0
SDS3	0	0.078	0.002	0.045	0.009	0	0.020	0	0	0	0.033	0	0	0.044	0.008	0.012	0	0	0	0.009	0.179	-0.007	0	0	0	0	0	0.101	0.059	0.045	0	0	0.057	0	0.028	0	0	0	0.121	0

SDS4	0	0	0	0.030	0	0	0.008	0.035	0	0.017	0	0.001	-0.034	0.021	0	0.075	0	0	0.255	0.176	0.042	-0.060	0	0	0	0	0.052	0.084	0.022	0.103	0	-0.041	0.074	0	0.022	-0.007	-0.010	0	0.038	0
SDS5	0	0	0	0	0.064	0.027	0	0	0.129	0	0	0	0.102	0	0	0	0.039	0	0.064	0	0	0	0	0	0	0.218	0	0	0	0	0.160	0.070	0	0	0	0	0	0	0	0.162
SDS6	-0.040	0	0	0	0.020	0	0	0	0.009	0	0	0	0	-0.013	0	-0.026	0	0	0.055	0	0	0.022	0	0	0.218	0	0	-0.022	0	0	0.067	0.017	0	0.087	-0.013	0.051	0.076	0	0	0
SDS7	0	0	0	0.028	0	0	0	0	0	0	0	0.014	0	0.011	0.066	0	0	0	-0.010	0	0.003	-0.061	0	0.052	0	0	0	0.100	0.140	0	0	-0.025	0.055	0	0	-0.022	0	-0.026	0.076	0
SDS8	-0.038	0	0	0	0	0	0	0	0	0	0	0.023	0	0.105	0.157	0.048	0	0	0	0	0.011	-0.065	0.101	0.084	0	-0.022	0.100	0	0.074	0.025	0	-0.027	0.016	0	0.031	0	0	0	0.061	0
SDS9	-0.018	0	-0.005	0	0	0	0	0	0	0.276	0.053	0.123	0	0	0.018	0.018	0	0.014	0	0.005	0	-0.022	0.059	0.022	0	0	0.140	0.074	0	0.115	0	0	0.064	0	0.083	-0.038	0	0	0.115	0.015
SDS10	0	0.023	0.012	0.037	0	0	0	0.174	0	0	0	0	0	0	0.013	0	0	0.020	0	0	0.068	0	0.045	0.103	0	0	0	0.025	0.115	0	0	0	0.167	0	0.056	0	0	0	0	0
SDS11	0	0	0	0	0.034	0	0	0	0	0	0	0	0.040	0	0	0	0.010	0	0.031	0	0	0.056	0	0	0.160	0.067	0	0	0	0	0	0.288	0	0.116	0	0	0.092	0.024	0	0.086
SDS12	0	0	0	0	0.039	0	0	0	0.018	0	0	0	0.010	0	0	0	0.021	-0.041	0.019	-0.023	0	0.054	0	-0.041	0.070	0.017	-0.025	-0.027	0	0	0.288	0	0	0.081	-0.028	0.078	0	0.046	0	0.085
SDS13	0.022	0.026	0.006	0.047	0	0	0	0.013	0.021	0	0.041	0	0	0	0	0	0	0	0	0.063	0.176	0	0.057	0.074	0	0	0.055	0.016	0.064	0.167	0	0	0	0	0.222	-0.008	0	0	0.046	0
SDS14	-0.005	0	0	0.050	0.040	0	0	0	0.040	0	0.014	0	0	0	0	0	0	0	0.087	0	0	0.052	0	0	0	0.087	0	0	0	0	0.116	0.081	0	0	0	0.068	0.125	0.168	0.037	0.219
SDS15	0	0	0.062	0.006	0	0.044	0	0.028	0	0.012	0.001	0	0	0	0	0	0	0.086	0	0.033	0.093	0	0.028	0.022	0	-0.013	0	0.031	0.083	0.056	0	-0.028	0.222	0	0	0	0	0	0.062	0
SDS16	0	0	0	0	0.067	0	0	0	0.007	-0.013	0	0	0	-0.018	-0.025	0	0	0	0.032	-0.003	0	0.004	0	-0.007	0	0.051	-0.022	0	-0.038	0	0	0.078	-0.008	0.068	0	0	0.079	0.174	-0.089	0
SDS17	0	0	0	0.002	0.069	0	0	0	0.006	0	0	0	0	0	0	0	0.018	0	0.016	0	0	0.045	0	-0.010	0	0.076	0	0	0	0	0.092	0	0	0.125	0	0.079	0	0.378	0	0.113
SDS18	0	0	0	0.018	0.044	0	0	0	0	0	0	0	0	0	0	0	0	0	0	0	0.005	0	0	0	0	0	-0.026	0	0	0	0.024	0.046	0	0.168	0	0.174	0.378	0	0	0.163
SDS19	0	0	0	0.052	0	0.016	0	0	0	0	0	0.077	0	0.043	0	0	0	0	0	0.094	0.007	-0.048	0.121	0.038	0	0	0.076	0.061	0.115	0	0	0	0.046	0.037	0.062	-0.089	0	0	0	0
SDS20	-0.033	0	0	0	0	0	0	0	0.048	0	0	0.049	0.028	0	0	0	0.041	0	0.059	0	0	0	0	0	0.162	0	0	0	0.015	0	0.086	0.085	0	0.219	0	0	0.113	0.163	0	0

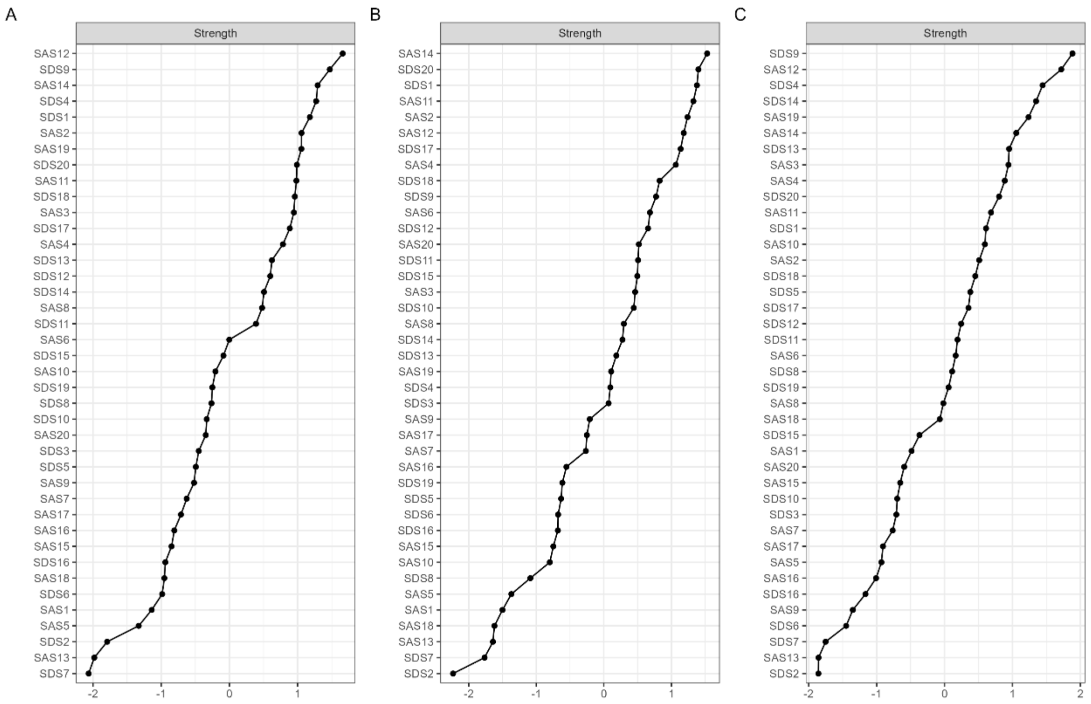

Figure S1. Centrality value. A, Standardized strength among all participants. B, Standardized strength of low-neuroticism group. C, Standardized strength of high-neuroticism group.

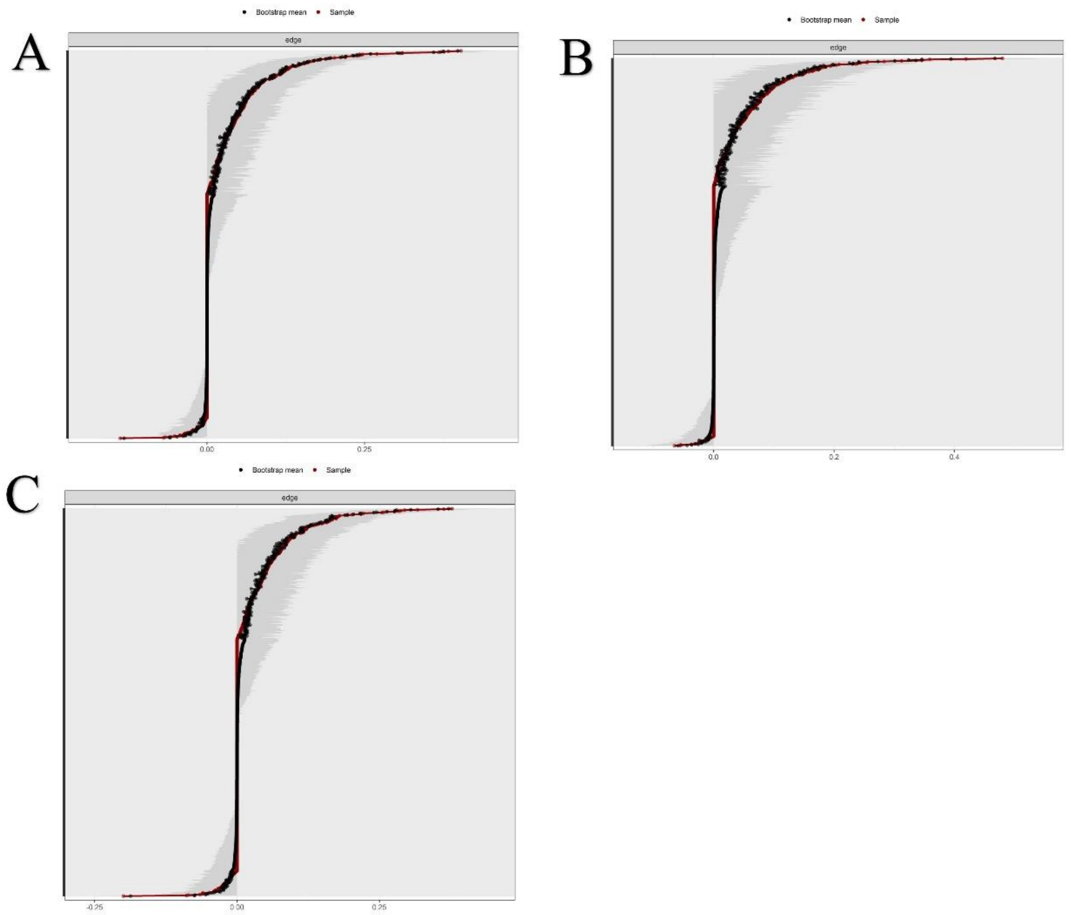

Figure S2. Nonparametric bootstrapped confidence intervals of estimated edges. The red line represents the estimated edge, while the dark area indicates the 95% bootstrap confidence interval. A indicates all participants. B indicates low-neuroticism group. C indicates high-neuroticism group.

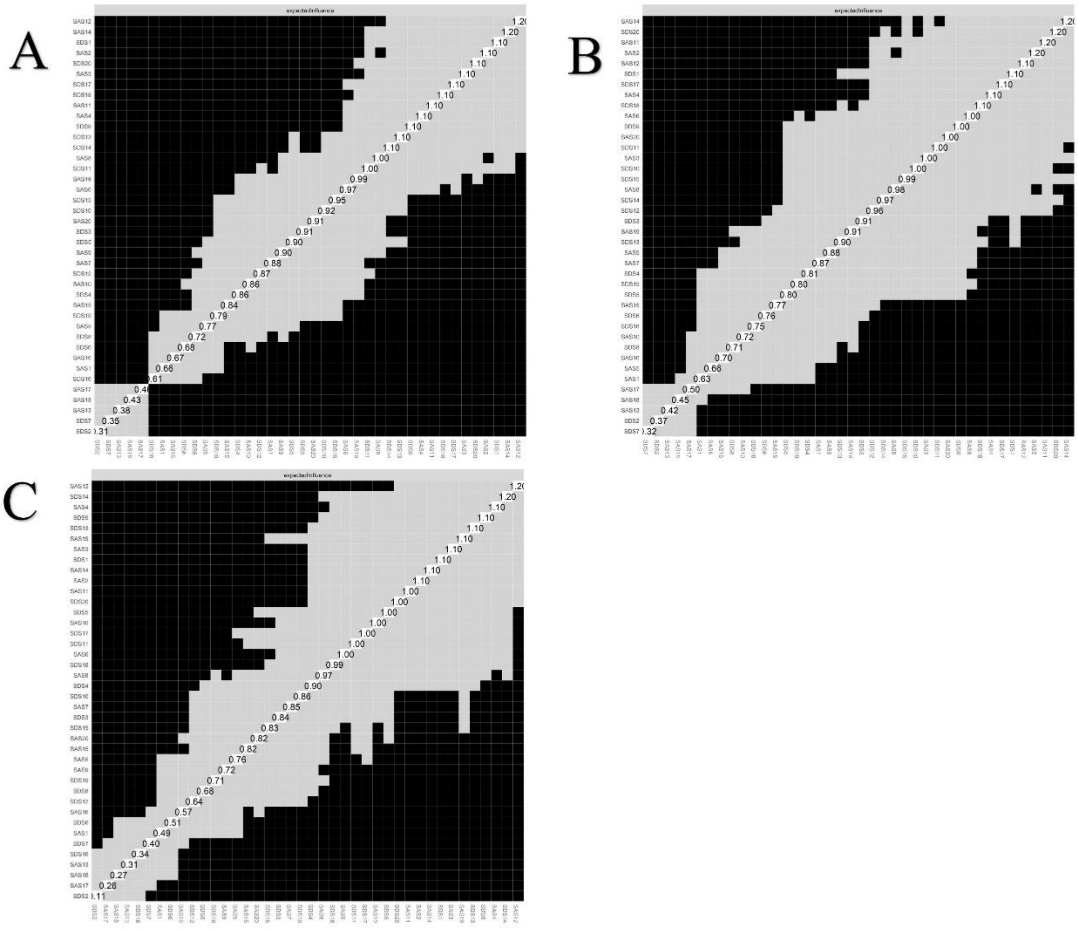

Figure S3. Nonparametric bootstrapped difference test for nodes. Grey boxes indicate no significant difference, whereas black boxes indicate a statistically significant difference ($p < 0.05$). Diagonal color and saturation represent the magnitude and direction of each estimated edge. A indicates all participants. B indicates low-neuroticism group. C indicates high-neuroticism group.

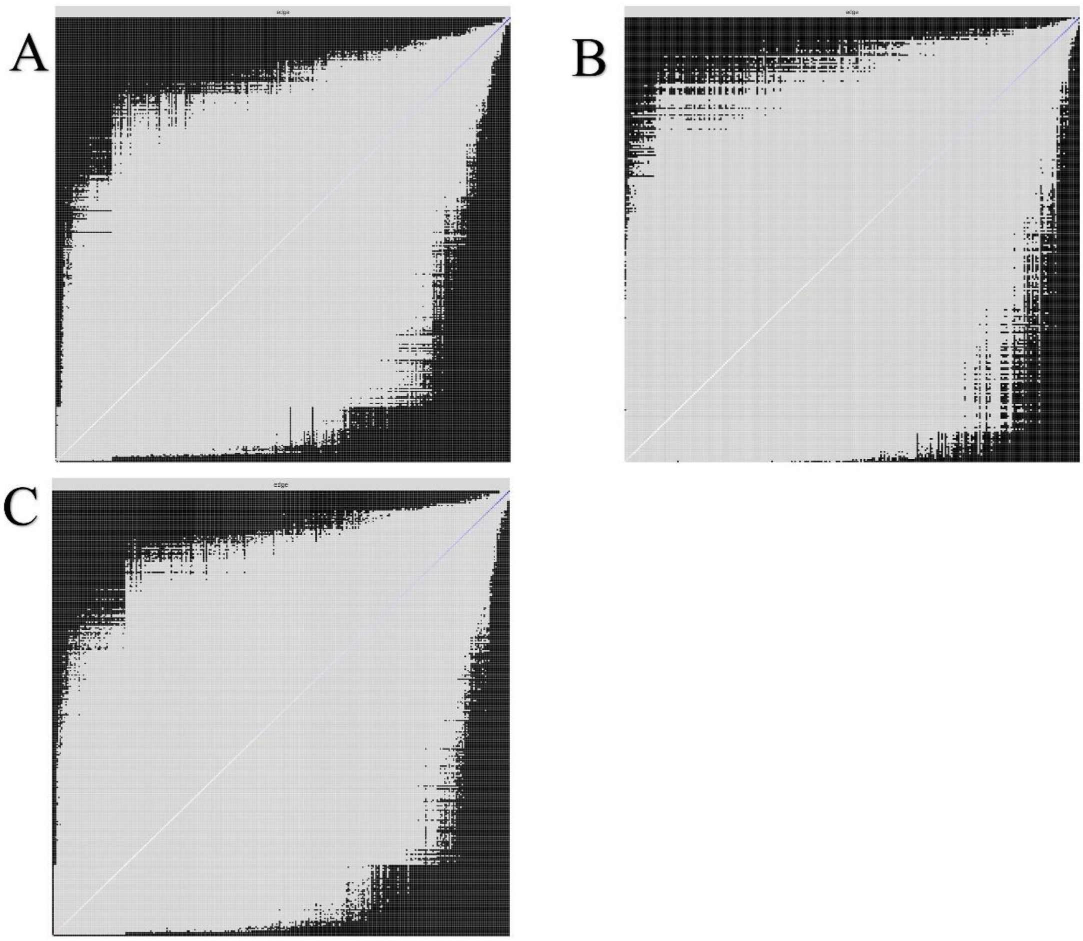

Figure S4. Bootstrapped stability test for edge-weight. The results of the bootstrapped difference tests ($\alpha = 0.05$) for edge-weights were shown in this figure. The colour of the boxes indicates whether edge-weights differ significantly from each other (i.e., black) or do not differ significantly (i.e., grey). The diagonal line indicates the strength of edge-weights, shifting from red (negative associations) to white (representing weaker edges) and ultimately blue (representing stronger edge-weights). A indicates all participants. B indicates low-neuroticism group. C indicates high-neuroticism group.

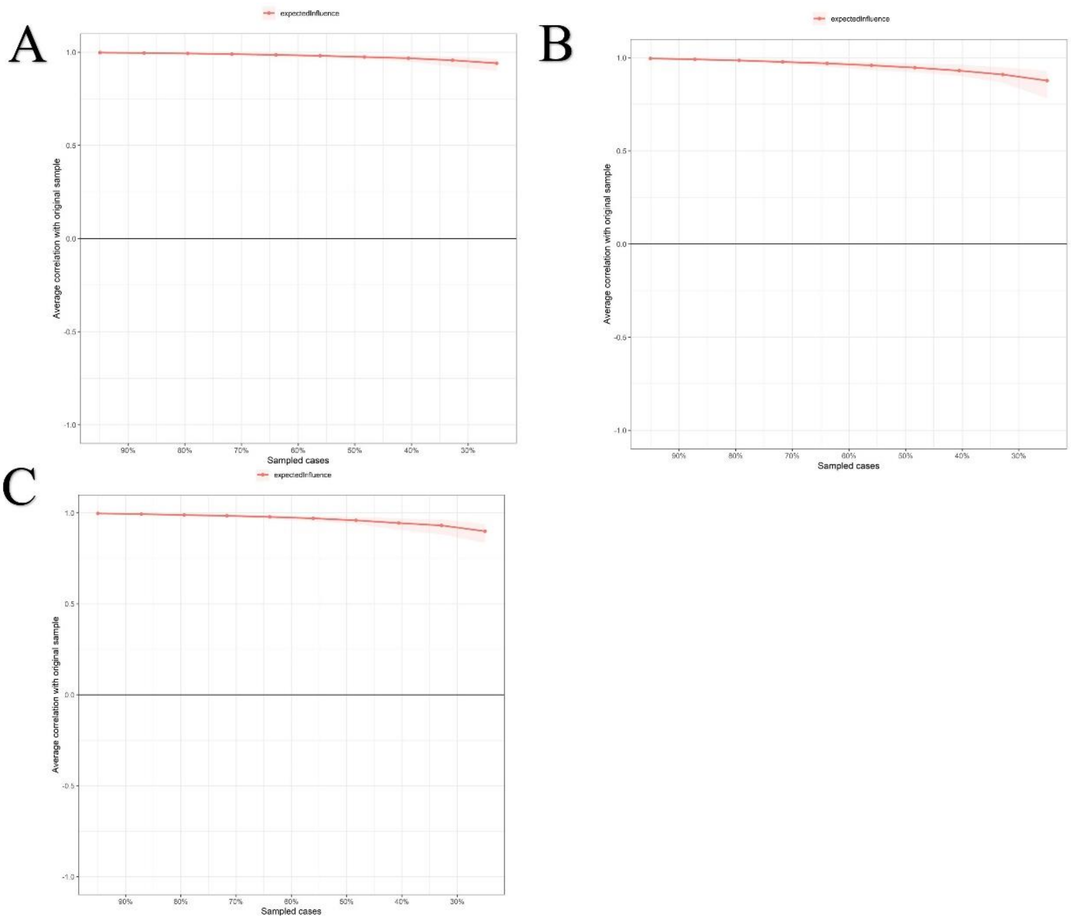

Figure S5. The x-axis indicates the percentage of cases of the original sample included at each step. The y-axis indicates the average correlations between the original network's centrality indices and the centrality indices from the networks that were re-estimated after excluding increasing percentages of cases. A indicates all participants. B indicates low-neuroticism group. C indicates high-neuroticism group.
